# Prenatal Treatment for Serious Neurological Sequelae of Congenital Toxoplasmosis: An Observational Prospective Cohort Study

**DOI:** 10.1371/journal.pmed.1000351

**Published:** 2010-10-12

**Authors:** Mario Cortina-Borja, Hooi Kuan Tan, Martine Wallon, Malgorzata Paul, Andrea Prusa, Wilma Buffolano, Gunilla Malm, Alison Salt, Katherine Freeman, Eskild Petersen, Ruth E. Gilbert, for The European Multicentre Study on Congenital Toxoplasmosis (EMSCOT)

**Affiliations:** 1 Centre for Pediatric Epidemiology and Biostatistics, UCL Institute of Child Health, London, United Kingdom; 2 Hospices Civils de Lyon, Service de Parasitologie, Hôpital de la Croix-Rousse, Lyon, France; 3 Department and Clinic of Tropical and Parasitic Diseases, University of Medical Sciences, Poznan, Poland; 4 Medical University of Vienna, Department of Paediatrics and Adolescent Medicine, Division of Paediatric Neonatology, Intensive Care and Neuropaediatrics, Vienna, Austria; 5 Perinatal Infection Unit, Department of Pediatrics, University of Naples Federico II, Naples, Italy; 6 Department of Clinical Science, Intervention and Technology, Karolinska Institutet, Division of Paediatrics, Karolinska University Hospital, Huddinge, Sweden; 7 Wolfson Centre, UCL Institute of Child Health, London, United Kingdom; 8 Department of Epidemiology and Population Health, Albert Einstein College of Medicine, Bronx, New York, United States of America; 9 Department of Infectious Diseases, Aarhus University Hospital, Skejby, Aarhus N., Denmark; University of Queensland Centre for Clinical Research, Australia

## Abstract

**Background:**

The effectiveness of prenatal treatment to prevent serious neurological sequelae (SNSD) of congenital toxoplasmosis is not known.

**Methods and Findings:**

Congenital toxoplasmosis was prospectively identified by universal prenatal or neonatal screening in 14 European centres and children were followed for a median of 4 years. We evaluated determinants of postnatal death or SNSD defined by one or more of functional neurological abnormalities, severe bilateral visual impairment, or pregnancy termination for confirmed congenital toxoplasmosis. Two-thirds of the cohort received prenatal treatment (189/293; 65%). 23/293 (8%) fetuses developed SNSD of which nine were pregnancy terminations. Prenatal treatment reduced the risk of SNSD. The odds ratio for prenatal treatment, adjusted for gestational age at maternal seroconversion, was 0.24 (95% Bayesian credible intervals 0.07–0.71). This effect was robust to most sensitivity analyses. The number of infected fetuses needed to be treated to prevent one case of SNSD was three (95% Bayesian credible intervals 2–15) after maternal seroconversion at 10 weeks, and 18 (9–75) at 30 weeks of gestation. Pyrimethamine-sulphonamide treatment did not reduce SNSD compared with spiramycin alone (adjusted odds ratio 0.78, 0.21–2.95). The proportion of live-born infants with intracranial lesions detected postnatally who developed SNSD was 31.0% (17.0%–38.1%).

**Conclusion:**

The finding that prenatal treatment reduced the risk of SNSD in infected fetuses should be interpreted with caution because of the low number of SNSD cases and uncertainty about the timing of maternal seroconversion. As these are observational data, policy decisions about screening require further evidence from a randomized trial of prenatal screening and from cost-effectiveness analyses that take into account the incidence and prevalence of maternal infection.

*Please see later in the article for the Editors' Summary*

## Abstract 2

An observational study by Ruth Gilbert and colleagues finds that prenatal treatment of congenital toxoplasmosis could substantially reduce the proportion of infected fetuses that develop serious neurological sequelae.

## Editors' Summary

**Background:**

Toxoplasmosis is a very common parasitic infection. People usually become infected with *Toxoplasma gondii*, the parasite that causes toxoplasmosis, by eating raw or undercooked meat that contains the parasite, but it can also be contracted by drinking unfiltered water or by handling cat litter. Most people with toxoplasmosis never know they have the disease. However, if a pregnant woman becomes infected with *T. gondii*, she can transmit the parasite to her unborn baby (fetus). Overall, about a quarter of women who catch toxoplasmosis during pregnancy transmit the parasite to their fetus. If transmission occurs early during pregnancy, the resultant “congenital toxoplasmosis” increases the risk of miscarriage and the risk of the baby being born with brain damage, epilepsy, deafness, blindness, or developmental problems (“serious neurological sequelae”). In the worst cases, babies may be born dead or die soon after birth. Congenital toxoplasmosis caught during the final third of pregnancy may not initially cause any health problems but eyesight problems often develop later in life.

**Why Was This Study Done?:**

Clinicians can find out if a woman has been infected with *T. gondii* during pregnancy by looking for parasite-specific antibodies (proteins made by the immune system that fight infections) in her blood. If the pattern of antibodies suggests a recent infection, the woman can be given spiramycin or pyrimethamine-sulfonamide, antibiotics that are thought to reduce the risk of transmission to the fetus and the severity of toxoplasmosis in infected fetuses. In some countries where toxoplasmosis is particularly common (for example, France), pregnant women are routinely screened for toxoplasmosis and treated with antibiotics if there are signs of recent infection. But is prenatal treatment an effective way to prevent the serious neurological sequelae or postnatal death (SNSD) associated with congenital toxoplasmosis? In this observational study, the researchers examine this question by studying a group of children identified as having congenital toxoplasmosis by prenatal or neonatal screening in six European countries. An observational study measures outcomes in a group of patients without trying to influence those outcomes by providing a specific treatment.

**What Did the Researchers Do and Find?:**

The researchers followed 293 children in whom congenital toxoplasmosis had been identified by prenatal screening (in France, Austria, and Italy) or by neonatal screening (in Denmark, Sweden, and Poland) for an average 4 years. Two-thirds of the children received prenatal treatment for toxoplasmosis and 23 fetuses (8% of the fetuses) developed SNSD; nine of these cases of SNSD were terminated during pregnancy. By comparing the number of cases of SNSD among children who received prenatal treatment with the number among children who did not receive prenatal treatment, the researchers estimate that prenatal treatment reduced the risk of SNSD by three-quarters. They also estimate that to prevent one case of SNSD after maternal infection at 10 weeks of pregnancy, it would be necessary to treat three fetuses with confirmed infection. To prevent one case of SNSD after maternal infection at 30 weeks of pregnancy, 18 fetuses would need to be treated. Finally, the researchers report that the effectiveness of pyrimethamine-sulfonamide and spiramycin (which is less toxic) was similar, and that a third of live-born infants with brain damage that was detected after birth subsequently developed SNSD.

**What Do These Findings Mean?:**

These findings suggest that prenatal treatment of congenital toxoplasmosis could substantially reduce the proportion of infected fetuses that develop SNDS and would be particularly effective in fetuses whose mothers acquired *T. gondii* during the first third of pregnancy. These findings should be interpreted with caution, however, because of the small number of affected fetuses in the study and because of uncertainty about the timing of maternal infection. Furthermore, these findings only relate to the relatively benign strain of *T. gondii* that predominates in Europe and North America; further studies are needed to test whether prenatal treatment is effective against the more virulent strains of the parasite that occur in South America. Finally, because this study is an observational study, its findings might reflect differences between the study participants other than whether or not they received prenatal treatment. These findings need to be confirmed in randomized controlled trials of prenatal screening, therefore, before any policy decisions are made about routine prenatal screening and treatment for congenital toxoplasmosis.

**Additional Information:**

Please access these Web sites via the online version of this summary at http://dx.doi.org/10.1371/journal.pmed.1000351.

- The US Centers for Disease Control and Prevention provides detailed information about all aspects of toxoplasmosis, including toxoplasmosis in pregnant women (in English and Spanish)
- The UK National Health Services Choices website has information for patients about toxoplasmosis and about the risks of toxoplasmosis during pregnancy
- KidsHealth, a resource maintained by the Nemours Foundation (a not-for-profit organization for children's health), provides information for parents about toxoplasmosis (in English and Spanish)
- Tommy's, a nonprofit organization that funds research on the health of babies, also has information on toxoplasmosis
- MedlinePlus provides links to other information on toxoplasmosis (in English and Spanish)
- EUROTOXO contains reports generated by a European consensus development project
- Uptodate provides information about toxoplasmosis and pregnancy

## Introduction

Congenital toxoplasmosis occurs when a woman first acquires *Toxoplasma gondii* infection during pregnancy. Infection can be acquired from oocysts ingested from contaminated soil or water, or tissue cysts from infected meat, but can only reliably be detected by seroconversion (change from negative to positive toxoplasma-specific antibodies) [1],[2]. Overall, the proportion of mothers who transmit infection to their fetus averages 25% but increases steeply with the gestational age at maternal seroconversion [3],[4]. Congenital toxoplasmosis leads to postnatal clinical manifestations of retinochoroiditis and/or intracranial lesions in one in six (17%) infected infants [3], and further eye lesions can appear at any age [5]. Less is known about the risk of neurological impairment, even though the main purpose of prenatal screening is to prevent serious neurological sequelae or death (SNSD) [3]. No prospective, comparative studies have evaluated the effectiveness of prenatal treatment for reducing SNSD.

We used variation in screening practices across Europe to determine the effect of prenatal treatment on SNSD. Our data derive from two prospective cohort studies in 14 centres in six European countries [6],.

## Methods

### Study Population

We studied infected fetuses that were prospectively identified by universal prenatal screening and newborn screening for congenital toxoplasmosis [4],[6]–[8]. Criteria for congenital toxoplasmosis are reported elsewhere [4],[6]. To avoid referral bias, we included only mothers or children in whom a positive screen test result preceded prenatal treatment or diagnostic investigations.

### Follow-Up

Infected fetuses were enrolled into the study after confirmation of a positive prenatal screening test in France, Austria, and Italy, or neonatal screening test in Denmark, Sweden, or Poland. Screening and treatment schedules are summarized in Tables 1 and 2. Information on clinical and laboratory findings and treatment were collected at the end of pregnancy, at 1 mo postnatally, and at every pediatric and ophthalmic examination for toxoplasmosis at approximately 6 and 12 mo, and then annually until at least 4 y of age [5],[6].

**Table 1:** Characteristics of universal prenatal screening protocols and patients in study centres.

Prenatal Screening	France	Austria	Italy	All Prenatal Screening
**Years of recruitment**	1996–1999	1996–2000	1996–2000	
**Centres**	Lyon, Paris, Marseille, Toulouse, Nice, Reims, Grenoble	Vienna	Milan, Naples	
**Screening and treatment schedules**				
Testing regimen	PN monthly retesting	PN + retesting at 12, 20, 32 wk	PN monthly (Milan) or 3-monthly (Naples) retesting	
First prenatal treatment	Spiramycin	P&S after 15 wk	Spiramycin	
***n*** ** Fetuses with congenital toxoplasmosis**	182	24	15	221
Percent treated prenatally	85	88	93	86
SNSD cases	2+9 terminations	1	2	14
**GASC (wk)**				
Median imputed GASC (IQR)	29.0 (23.0–33.1)	18.8 (17.3–25.9)	18.5 (16.2–20.4)	27.3 (19.7–32.4)
Median interval (IQR)	4.0 (5.0–8.0)	17.1 (11.9–20.5)	12.6 (9.1–14.1)	5.4 (4.1–11.4)
**Prenatal treatment**				
Total treated	135	21	14	189
Percent spiramycin as first treatment	88	10	93	79
Median imputed GASC to treatment interval, wk (IQR)	2.9 (2.3–4.0)	11.0 (7.9–13.0)	5.9, (4.9–7.0)	3.1 (2.6–5.7)
**Follow-up, y (IQR)**	4.0 (3.2–4.8)	4.3 (4.0–5.1)	4.0 (1.0–4.1)	4.0 (3.2–4.7)

**Table 2 pmed-1000351-t002:** Characteristics of neonatal screening protocols and patients in study centres.

Neonatal Screening	Sweden	Poland	Denmark	Denmark 1992–1996	All Neonatal Screening
**Years of recruitment**	1997–1998	1996–2000	1997–2000	1992–1996	
**Centres**	Stockholm, South Sweden	Poznan	Copenhagen	National study	
**Screening and treatment schedules**					
Testing regimen	Neo, IgG_R_ and IgM	Neo, IgM and IgA	Neo, IgM and IgA	Neo, IgG_R_ and IgM	
First prenatal treatment					
***n*** ** Fetuses with congenital toxoplasmosis**	3	29	14	26	72
Percent treated prenatally	0	0	0	0	0
SNSD cases	1	4	1	3	9
**GASC (wk)**					
Median imputed GASC (IQR)	27.9 (27.3–27.9)	27.6 (26.9–29.4)	26.9 (26.9–27.5)	27.5 (26.8–29.6)	27.3 (26.9–29.2)
Median interval (IQR)	28.1 (26.1–30.6)	38.4 (36.4–40.6)	40.0 (40.0–40.0)	31.1 (29.2–32.4)	36.4 (31.5–40.0)
**Follow-up, y (IQR)**	3.9 (3.8–4.6)	4.1 (3.9–4.3)	3.5 (3.2–3.9)	6.3 (4.9–6.4)	4.2 (3.5–6.1)

IgG_R_ involves detection of IgG seroconversion by comparing neonatal sample with prenatal booking sample from mother.

Neo, neonatal screening based on detection of specific antibodies in Guthrie card bloodspots; P&S, pyrimethamine and sulphonamide; PN, prenatal screening.

Details recorded during pregnancy included the date of the first abnormal or last normal fetal ultrasound scan, the results of PCR testing of amniotic fluid for toxoplasma DNA, autopsy findings for terminated fetuses, and the start and end dates of any prenatal treatment. Mothers of infants detected in neonatal screening centres were not treated. The variation between centres in the delay before starting prenatal treatment after maternal seroconversion and in the use of spiramycin or pyrimethamine-sulphonamide combinations as first-line treatment is shown in Table 1 for infected babies identified by universal prenatal screening and in Table 2 for babies identified by neonatal screening, and reported in detail elsewhere [6]. All centres treated infected babies postnatally, but there were minor differences in the use of pyrimethamine-sulphonamide alone or alternating with spiramycin [6].

### Serious Neurological Sequelae

The primary outcome was serious neurological sequelae or death (SNSD), a composite outcome, comprising a pediatric report at any age of microcephaly, insertion of intraventricular shunt, an abnormal or suspicious neurodevelopmental examination that resulted in referral to a specialist, seizures during infancy or at an older age that required anticonvulsant treatment, severe bilateral visual impairment (visual acuity of Snellen 6/60 or less in both eyes assessed after 3 y), cerebral palsy, or death from any cause before 2 y of age including termination of pregnancy [9]. The consistency of SNSD findings was checked through multiple assessments. We did not require evidence that SNSD was attributable to congenital toxoplasmosis as this difficult clinical judgment could have biased inclusion of cases. Fetuses terminated for congenital toxoplasmosis were assumed to have SNSD, owing to long-standing policy discouraging termination unless there is evidence of intracranial lesions or other adverse sequelae affecting the fetus [10]–[12]. This assumption allows a conservative analysis of the effect of prenatal treatment as women were always treated before termination. Sensitivity analyses explored the increase in treatment effect after assuming no SNSD for terminated fetuses.

### Gestational Age at Maternal Seroconversion

In order to avoid well-known biases introduced by imputing a covariate as an unadjusted midpoint for interval values of the gestational age at maternal seroconversion (GASC), we imputed values for GASC using all the serological information available to us [13]. For women screened prenatally, we imputed seroconversion at the midpoint between the last negative and first positive immunoglobulin M (IgM) tests, unless the woman was IgG negative at the first IgM positive test, when seroconversion was assumed to be 14 d beforehand. This method was derived from a previous analysis of a cohort from Lyon, France [14]–[16], and has been confirmed with subsequent cohorts [6].

For babies identified by neonatal screening, maternal seroconversion was based on an adjusted midpoint between conception, or in Sweden and Denmark 1992–1996, between the first prenatal booking sample and birth, using a previously reported log linear regression model [5]. The model was derived in the cohort of prenatal screened women and used ranked IgG titre and IgM status at the first neonatal test as predictors of GASC. These modifications shifted the imputed GASC to the right of the midpoint. This shift is consistent with evidence that the probability of a positive IgM result in an infected baby increases with GASC and that increased IgG titre is associated with recent infection [17],[18].

### Analyses

All live births had at least one pediatric assessment during infancy (up till 12 mo old). Children with a normal pediatric examination during infancy who were subsequently lost to follow-up were assumed to have no SNSD [19].

**Figure 1:**
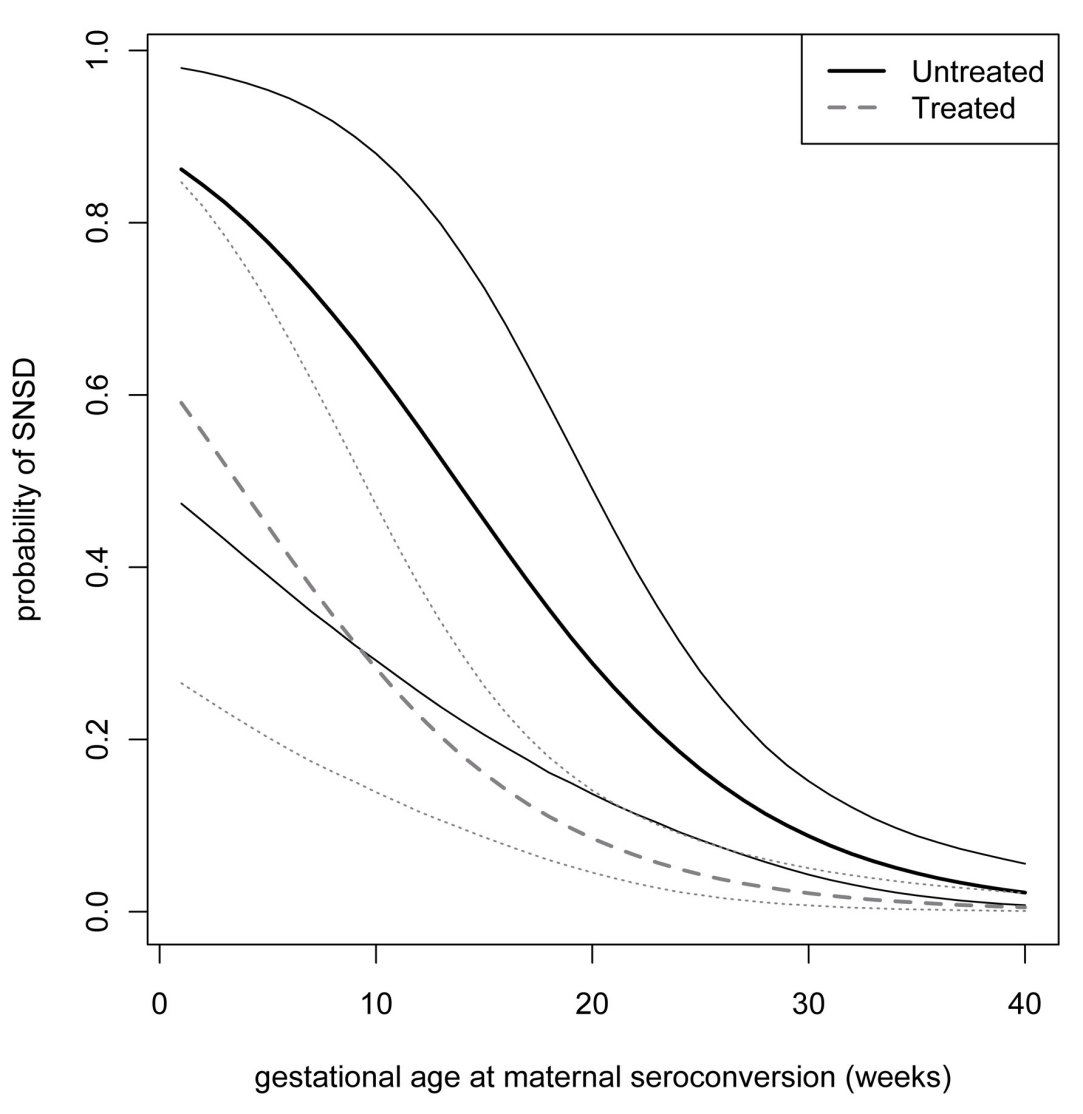
Probability of SNSD. Probability of SNSD according to imputed gestational age at seroconversion and 95% Bayesian credible limits: dotted lines denote treated pregnancies; solid lines denote untreated pregnancies.

We used WinBUGS version 1.4.3 to estimate odds ratios for the effect of prenatal exposures on SNSD. All models were adjusted for GASC [5]. The Bayesian framework was chosen because there were numerical problems, caused by the low event rate and the relatively small sample size, in the optimization algorithms required to find maximum likelihood estimates, which meant that the asymptotic normal approximation required to construct confidence intervals was unreliable. The Bayesian framework avoids relying on the large-sample normal approximation and has the advantage of evaluating the uncertainty from derived, nonlinear quantities without further using permutational or resampling methods. An example of this type of propagation of errors can be seen in the credible intervals presented in Figure 1. Our inferences were based on 95% Bayesian credible intervals (BCIs) referring to the posterior distributions of the models' parameters and derived quantities. All models were adjusted for gestational age at seroconversion, as previously reported [5].

The posterior probability distributions of SNSD for each level of exposure were calculated using Markov Chain Monte Carlo (MCMC) iterations. We used noninformative priors in two mixing Markov chains with different starting values and allowed 1,000 MCMC burn-in iterations; estimates of all posterior distributions were based on 10,000 realizations of each chain. A linear transformation was applied to the imputed values of gestational age at maternal seroconversion by centering around its mean; this improved the MCMC convergence, assessed by the method proposed by Brooks and Gelman [20], and made the autocorrelation functions close to zero. Inclusion of a quadratic term for GASC did not improve the goodness of fit according to the Deviance Information Criterion [21]. This method produced similar results for the effect of prenatal treatment to a previously used maximum likelihood estimation procedure [6].

Sensitivity analyses were used to determine the robustness of the findings to different assumptions. First we explored under-reporting of terminations by excluding data from Austria and Italy, as no terminations were reported for these centres. We also determined whether adding 18 unreported terminated fetuses to the analyses would alter the treatment effect. The 18 unreported fetuses had the same characteristics (GASC and prenatal treatment delay) as the nine terminated fetuses included in the study. Second, we estimated the effect of treatment under the extreme assumption that all terminated fetuses would have been born alive and would have developed normally. We revised this analysis assuming that the three fetuses with normal fetal ultrasound scans and no evidence of disseminated infection would have developed normally, even though two fetuses had their last scan before 21 wk of gestation. Third, we reclassified three live-born, untreated children with SNSD but without intracranial calcification as no SNSD. This reclassification restricted the outcome of SNSD to cases almost certainly attributable to congenital toxoplasmosis. Fourth, we explored potential error in the imputed gestational age at maternal seroconversion for children identified by neonatal screening by assuming seroconversion 1 mo earlier.

We calculated the number of women needed to be treated (NNT) to prevent one case of SNSD as a clinically meaningful measure for counseling women about the absolute difference in the probability of their child developing SNSD with and without treatment. We used the posterior probability distributions and 95% credible interval calculated from the regression model to estimate the NNT for women after a positive prenatal diagnosis (i.e., the number of women with an infected fetus who need to be treated). To estimate the NNT for women without prenatal diagnosis, we multiplied the difference in probability with the estimated risk of mother to child transmission of toxoplasmosis derived from a meta-analysis of all available cohort studies [3]. This simple estimation was based on the assumption that prenatal treatment has no effect on mother to child transmission. We did not take into account random error in the estimation of mother to child transmission according to GASC.

To inform prognostic counseling for parents of live-born babies, we determined the probability of SNSD according to an exclusive hierarchy of postnatal clinical manifestations (ventricular dilatation and/or intracranial calcification, lymphadenopathy or hepatosplenomegaly, and/or retinochoroiditis) that would be apparent after pediatric examination, ophthalmoscopy, and postnatal cranial ultrasound completed by 6 mo postnatal age [1]. These analyses excluded the Danish cohort of 1992 to 1996 (*n* = 26) owing to missing data for these early manifestations [7].

Research ethics approval was granted in countries where screening was offered as part of a research study [7],[22],[23], but was not required where screening was routine practice.

## Results

### Study Population and Neurological Sequelae

The combined cohorts comprised 293 infected fetuses, of whom 284 were born alive. 23 fetuses consisting of nine terminated pregnancies and 14 live-born children died or were classified as having serious neurological sequelae (Tables 1–4).The distribution of GASC and the intervals from which GASC was imputed are summarized in Tables 1 and 2, and Figures S1 and S2. All live-born children had at least one pediatric examination. The last examination was before 6 mo of age for 6/284 (2%) children and between 9 and 12 mo for a further six children. 238/284 (84%) children were followed beyond 2 y of age. Overall, the median duration of follow-up was 4.05 y (interquartile range [IQR] 3.21–4.88). Follow-up was longer for 26 children enrolled in the Danish cohort in 1992–1996 (6.3 y, IQR 4.9–6.4) (Table 2), but did not differ significantly between live-born children whose mothers were treated (4.05, IQR 3.25–4.76) compared with not treated (4.13, IQR 3.17–5.19, Wilcoxon-Mann-Whitney test *p* = 0.39).

**Table 3 pmed-1000351-t003:** Characteristics of live births with congenital toxoplasmosis and SNSD.

Characteristic	Neonatal screened (*n* = 9)	Prenatal screened (*n* = 5)
**Prenatal and birth**		
Treated prenatally	0	4
Abnormal fetal ultrasound	2 (4 NR)	3
Gestational age at birth	26–42 wk	34–39
**Clinical manifestations before ≤6 mo**		
Intracranial lesions (postnatal cranial ultrasound)	7	4
Retinochoroidits	5 (1 NR)	4
Lymphadenopathy/hepatosplenomegaly	2 (3 NR)	3
**Outcomes reported during follow-up**		
Death before 2 y	3	1
Microcephaly, seizures, or shunt required	5	4
Cerebral palsy or abnormal neurological development^a^	5	3
Ocular complications^b^	5	5
Blindness (≤6/60)	1 (4 NR)	2 (1 NR)

a Abnormal or suspicious neurological examination leading to referral to a specialist.

b Ocular microphthalmia (*n* among all live births with SNSD = 5), visual opacities (*n* = 4), cataract (*n* = 2), strabismus (*n* = 4).

NR, not reported.

**Table 4:** Characteristics of fetuses terminated with congenital toxoplasmosis.

Characteristic	Abnormal Fetal Ultrasound^a^ (*n* = 5)	Normal Fetal Ultrasound (*n* = 4)
Gestational age at maternal seroconversion (wk)	10–17	8–13
Gestational age at first abnormal or last normal fetal ultrasound (wk)	21–30	11–23
Termination of pregnancy (wk of gestation)	22–33	12–24
Autopsy findings (*n* fetuses affected)		
Macroscopic examination not reported	1	3
Intracranial abnormalities	1	—
Myocarditis, pneumonitis, disseminated disease	1	1
Nil abnormal found	2	—

All fetuses had positive PCR detection of *T. gondii* DNA in amniotic fluid (*n* = 8) or culture based on mouse inoculation of fetal products (*n* = 3).

a Intracranial calcification or ventricular dilatation on fetal ultrasound scan.

Of the 221 women identified by prenatal screening, 86% (189/221) received prenatal treatment, most (71%, 134/189) within 5 wk of the imputed date of maternal seroconversion (Table 4). Untreated women (*n* = 32) identified by prenatal screening had their first positive serological test at a median gestational age of 38.6 wk (IQR 37.4–40.0 wk). None of the 72 women with infected live-born infants identified by neonatal screening received prenatal treatment (Table 2).

The characteristics of the 23 fetuses with SNSD are summarized in Tables 3 and 4. Among the 14 live births, most (11/14) had SNSD first detected in the first 6 mo of life. Three untreated, live-born children had no intracranial lesions detected postnatally. Two of the three were identified by neonatal screening. One had no ocular manifestation but was classified with SNSD because the baby died at 3 mo from staphylococcal septicemia. The other two had ocular signs: one had retinochoroiditis and strabismus and the other had a cataract. Less information was available for the nine terminated fetuses (Table 4): six had intracranial abnormalities on fetal ultrasound and/or macroscopic abnormalities at autopsy.

### Characteristics Associated with Serious Neurological Sequelae

Three prenatal factors strongly predicted serious neurological sequelae: the gestational age at maternal seroconversion, prenatal treatment, and an abnormal fetal ultrasound of the brain (Table 4). The odds of SNSD, adjusted for prenatal treatment, decreased by 13% (95% BCI 7%–19%) for every week increase in gestational age at maternal seroconversion (odds ratio 0.866, 95% BCI 0.806–0.925) (Table 5).

**Table 5:** Prenatal characteristics associated with SNSD.

Characteristic	Total Infected Fetuses (*n*)	Fetuses with SNSD (*n*)		Odds Ratio for SNSD^a^	95% BCI	Estimated proportion (%) with SNSD^a^	95% BCI
		Terminated	Live Birth				
**All children**	293	9	14			7.73	(5.11–11.21)
**Imputed GASC**							
Per week of gestation				0.904	0.855–0.953		
≤20 wk	57	9	4	Ref.	—	22.81	13.00–34.29
>20 wk	236	0	10	0.290	0.148–0.520	4.12	2.03–7.12
**Screening centre**							
Neonatal	72	0	9	Ref.	—	14.95	7.42–25.69
Prenatal	221	9	5	0.170	0.046–0.534	2.90	1.04–6.12
**Country**							
France	182	9	2	Ref.	—	3.18	1.14–6.75
Italy/Austria	39	0	3	0.444	0.083–1.784	1.42	0.23–5.56
Scandinavia	43	0	5	4.798	1.202–19.24	13.58	5.05–27.29
Poland	29	0	4	5.981	1.28–26.94	16.40	5.21–34.62
**Gestation at birth** b							
Term	246	0	10	Ref.	—	3.82	1.91–6.79
Preterm	38	9	4	2.679	0.653–9.195	9.62	2.87–22.08
**Gender** b		5 unknown					
Girl	130	2	5	Ref.	—	3.46	1.24–7.66
Boy	154	2	9	1.625	0.524–5.381	5.51	2.64–9.89
**Fetal ultrasound** c							
Normal	204	4	2	Ref.	—	0.59	0.71–2.28
Any abnormality	14	5	3	120	7.035–6400	42.63	6.09–90.16
**Prenatal treatment**							
Untreated	104	0	10	Ref.	—	11.99	6.02–20.82
Treated	189	9	4	0.236	0.071–0.708	3.11	1.21–6.47
*Type of treatment* d							
Spiramycin only	87	5	2	Ref.	—	2.37–	0.48–7.62
Any P&S treatment	102	4	2	0.777	0.204–2.849	1.84	0.43–5.39
*Treatment delay after seroconversion* d							
≤35 d	134	6	1	Ref.	—	0.45	0.02–2.54
>35 d	55	3	3	0.7581	0.190–2.862	2.83	0.34–11.00

a Adjusted for gestational age at maternal seroconversion.

b Sample restricted to live births.

c Sample restricted to mother-child pairs in prenatal centres who had fetal ultrasound (218/221).

d Sample restricted to those prescribed prenatal treatment.

P&S, pyrimethamine and sulphonamide treatment.

Prenatal treatment substantially reduced the risk of serious neurological sequelae. The adjusted odds ratio for any prenatal treatment compared with no treatment was 0.236 (95% BCI 0.071–0.708), and the average risk difference between treated and untreated mothers was 8.7% (2.0%–18.1%) (Table 5). In infected fetuses, the absolute risk difference between SNSD in treated and untreated pregnancies declined steeply with the gestational age at maternal seroconversion (Figure 1). After maternal seroconversion at 10 wk of gestation, the estimated risk of SNSD in fetuses of treated women was 25.7% (12.9%–43.0%) and in untreated women 60.0% (27.6%–85.9%). The risk difference was 33.3% (6.9%–56.1%), and the NNT of mothers with infected fetuses to prevent one case of SNSD was 3 (2–15). After seroconversion at 20 wk, the risk difference was 18.5 (3.6–38.3, NNT 6 [3]–[28]), and after seroconversion at 30 wk, the risk difference was 5.7 (1.3–11.5, NNT 18 [9–75]). The NNT to prevent one case of SNSD for women who do not know whether their fetus is infected or not was estimated to be 28 (17–132) after seroconversion at 10 wk of gestation; 20 (10–99) at 20 wk, the lowest point; 32 (16–137) at 30 wk; and 51 (23–201) after seroconversion at 35 wk of gestation. These estimates apply only to women with confirmed seroconversion.

Table 6 shows that sensitivity analyses did not alter the finding of a significant treatment effect at the 5% level, except when three untreated live-born children with SNSD but no intracranial lesions were reclassified without SNSD. The adjusted odds ratio for any prenatal treatment compared with none was reduced in analyses that excluded Austria and Italy and when terminated fetuses were reclassified without SNSD (Table 6). The addition of a further 18 fictitious, unreported pregnancy terminations with SNSD marginally reduced the treatment effect. Assuming maternal seroconversion 1 mo earlier in cases identified by neonatal screening also increased the odds ratio, but the upper 95% credible interval still excluded 1.0 (Table 6).

**Table 6:** Sensitivity analyses (odds ratios for SNSD and 95% BCIs).

Rationale for Sensitivity Analysis	Infected Fetuses *(n)*	SNSD (*n*)	Univariable Model		Multivariable Model			
			GASC		Adjusted GASC		Prenatal Treatment Versus None	
			OR	95% BCI	OR	95% BCI	OR	95% BCI
**Analytic approach**								
Bayesian estimate	293	23	0.904	(0.855–0.953)	0.866	(0.806–0.925)	0.236	(0.071–0.708)
Ordinary logistic regression	293	23	0.903	(0.857–0.953)	0.870	(0.814–0.930)	0.248	(0.081–0.756)
**Varying outcome status**								
**Under-reporting of terminations**								
Excluding Italy and Austria	254	11	0.882	(0.829–0.936)	0.819	(0.741–0.889)	0.123	(0.025–0.461)
Addition of 18 fictitious unreported terminations	311	41	0.849	(0.806–0.889)	0.817	(0.763–0.867)	0.294	(0.096–0.850)
**Assumption of adverse outcome in terminated fetuses**								
No terminations have SNSD	293	14	0.999	0.931–1.082)	0.948	(0.870–1.035)	0.136	(0.029–0.498)
Terminations with normal fetal ultrasound and no disseminated disease have no SNSD	293	20	0.926	(0.875–0.980)	0.890	(0.829–0.951)	0.226	(0.067–0.692)
**Outcomes not attributable to congenital toxoplasmosis**								
Three live births without intracranial lesions reclassified as not SNSD	293	20	0.889	(0.838–0.941)	0.865	(0.804–0.923)	0.367	(0.104–1.230)
One live birth without intracranial lesions and no ocular signs reclassified as not SNSD	293	22	0.902	(0.853–0.952)	0.871	(0.811–0.927)	0.287	(0.085–0.858)
**Timing of GASC**								
Imputed GASC placed 1 mo earlier in neonatal screened.	293	23	0.883	0.830–0.935)	0.861	(0.798–0.919)	0.356	(0.125–0.992)

OR, odds ratio.

The nine terminated pregnancies in France made up most of the SNSD cases that seroconverted in the first trimester (Figures S1 and S2). As a result, the effect of GASC on SNSD diminished and was no longer significant at the 5% level when all terminations were reclassified without SNSD (Table 6).

Among treated women, we found no evidence that delayed timing of treatment increased the proportion of fetuses with SNSD. However, the power to detect such an effect was limited. Moreover, most of the early treated fetuses with SNSD were pregnancy terminations (Table 4). There was no evidence that pyrimethamine-sulphonamide treatment was clinically or statistically more beneficial than spiramycin alone, although there was limited power to detect an effect at the 5% level (Table 5).

Fetal ultrasound abnormality was associated with SNSD (adjusted odds ratio 120, 7.04–6400), partly because this was a criterion for pregnancy termination, and hence classification with SNSD. The earliest gestational age at detection of intracranial abnormality on fetal ultrasound was 21 wk in five terminated fetuses and 26 wk in five live-born fetuses (three with SNSD). All five live-born fetuses with intracranial abnormality on fetal ultrasound had intracranial calcification and/or ventricular dilatation on postnatal cranial ultrasound (specificity 178/178, 100%), but few babies with abnormal postnatal scans had intracranial abnormalities reported on fetal ultrasound (5/18, sensitivity 28%).

Table 7 shows the distribution of serious neurological sequelae according to postnatal clinical manifestations detected in early infancy in live-born children with congenital toxoplasmosis. The proportion of children with serious neurological sequelae was higher among those with intracranial lesions detected by postnatal cranial ultrasound scan (30.2%, 95% BCI 13.7%–46.6%) than in those with no intracranial lesions (1.0%, 0.0%–2.3%).

**Table 7:** Postnatal clinical manifestations detected in early infancy and probability (%) of SNSD.

Postnatal Clinical Manifestations	Postnatal Clinical Manifestations Detected at ≤6 mo Old	
	SNSD	Total Infected
	*n* = 11	*n* = 258^a^
All three signs (brain/eye/LHS)	4	4
Brain + eye	3	7
Brain + LHS	1	4
Brain only	1	15
Total with brain lesions	9	30
Proportion with SNSD (95% BCI)	30.2% (13.7%–46.6%)	
Eye + LHS	0	1
Eye only	0	13
LHS only	0	6
None	2	208
Total without brain lesions	2	228
Proportion with SNSD (95% BCI)	1.0% (0.0%–2.3%)	

Brain, intracranial calcification or ventricular dilatation detected on postnatal cranial ultrasound examination; eye, retinochoroiditis; LHS, lymphadenopathy or hepatosplenomegaly.

a Sample excludes Danish cohort recruited 1992–1996.

## Discussion

Prenatal treatment substantially reduced the proportion of infected fetuses who developed SNSD. We found no evidence that a pyrimethamine-sulphonamide combination was more effective than spiramycin, which is less toxic [24]. Among infected fetuses, the difference in the proportion of treated and untreated fetuses with SNSD was highest when maternal infection was acquired during the first trimester. These findings should be interpreted with caution because of the low number of the SNSD cases and the uncertainty about the timing of maternal seroconversion. Abnormalities on fetal ultrasound, and intracranial abnormalities detected by cranial ultrasound after birth, were important prognostic markers for SNSD.

This is the first prospective cohort study, to our knowledge, to report the effect of prenatal treatment on serious neurological sequelae in fetuses with congenital toxoplasmosis. We minimized selective inclusion of pregnancies with complications ensuring that universal screening tests preceded prenatal treatment or diagnostic investigations for fetal infection status or abnormalities. Long-term follow-up and repeated pediatric assessments made it possible to ensure that, for the live births at least, SNSD were confirmed. In a retrospective study, Foulon et al. reported a similarly large effect of prenatal treatment on intracranial lesions and/or neurological sequelae detected up to 12 mo of age, but these results could have been explained by referral of untreated women with pregnancy complications to fetal medicine centres [25],[26].

Weaknesses of the study relate to selection biases inherent in observational studies. First, pregnancies terminated for fetal infection may not have been reported to the study [27], but sensitivity analyses that assumed twice as many treated women had “unreported” terminations did not nullify the treatment effect. Second, we did not include stillbirths in the analysis as these rarely have a definitive diagnosis of congenital toxoplasmosis [28], which could have underestimated the benefits of prenatal treatment if treatment led to the survival of fetuses with SNSD who would have miscarried or been stillborn in untreated women. Third, our method for estimating the gestational age at maternal seroconversion may have overestimated precision and could have introduced bias [13]. Fourth, we assumed that all SNSD outcomes were attributable to congenital toxoplasmosis. Reclassification of three children with SNSD but without intracranial lesions to non-SNSD rendered the effect of prenatal treatment nonsignificant at the 5% level. Fifth, adverse outcomes were limited to serious manifestations that were evident on pediatric examination in the early childhood years. Overall, these potential biases act in different directions and are likely to only partly account for the strong treatment effect observed. However, they may alter the magnitude of the effect, which can only be reliably determined by a large randomized controlled trial.

### Implications for Practice

The benefits of prenatal treatment are high for women with a positive prenatal diagnosis for congenital toxoplasmosis. The NNT to prevent one case of SNSD varies from three to 18 depending on the gestational age at maternal seroconversion. As fetal diagnosis carries a risk of fetal loss due to amniocentesis, some women may prefer to trade a lower chance of benefit in order to avoid amniocentesis, provided a treatment such as spiramycin is used, which has no serious side effects [24]. The lowest estimate of the number of women with no prenatal diagnosis and unknown congenital infection status of their fetus who need to be treated to prevent one case of SNSD was 20 (ten to 99) after maternal seroconversion at 20 wk of pregnancy, rising to 28 (17 to 132) after seroconversion at 10 wk and 51 (23, 201) at 35 wk of gestation. These estimates apply only to women with confirmed seroconversion. The NNT would be much higher for women whose infection is diagnosed by tests for recent infection such as a rising titre, or low IgG avidity, as most of these women would have acquired infection before conception [4],[29].

Termination of pregnancy should be limited to fetuses with abnormal intracranial ultrasound findings, otherwise the vast majority of terminations would involve unaffected fetuses; this means deferring termination until 22 wk or later as ultrasound abnormalities do not develop until 21 wk of gestation at the earliest [30]. Termination at this late gestation involves feticide and is unlikely to be acceptable to many women or clinicians.

In terms of the type of treatment, our findings add to previous comparative studies, which have consistently found no evidence that pyrimethamine-sulphonamide combinations are more effective than the less toxic alternative of spiramycin [3],[4],[6],[15],[16],[25]. However, our study lacked power to detect an effect on SNSD.

Whether the benefits of prenatal treatment translate into an effective prenatal screening program remains to be determined by a randomised controlled trial of prenatal screening. In the meantime, cost-effectiveness analyses that take into account regional variation in the prevalence of susceptible women, the incidence of maternal infection, the timing, uptake, and accuracy of repeated screening tests to detect maternal seroconversion, and the timing of prenatal treatment, could provide valuable information for policy makers and for research funders contemplating investment in a large trial.

Finally, our results relate to the relatively benign type II strain of *T. gondii*, which predominates in Europe and North America. Trials are urgently needed to determine the most effective timing and type of prenatal treatment for the more virulent parasite strains that predominate in South America [31].

## Supporting Information

Figure S1 Interval for gestational age at seroconversion for pregnancies affected by SNSD. The vertical lines show the GASC interval for each pregnancy affected by SNSD (*n* = 23). Colours denote country of birth: red, France; yellow, Italy; light green, Austria; dark green, Poland; light blue, Denmark 1997–2000; purple, Denmark 1992–1996; pink, Sweden.
(0.01 MB PDF)

Figure S2 Interval for gestational age at seroconversion for unaffected pregnancies. The vertical lines show the GASC interval for each unaffected pregnancy (*n* = 270). Colour codes as for Figure S1.
(0.01 MB PDF)

## Abbreviations

BCI: Bayesian credible interval
GASC: gestational age at maternal seroconversion
Ig: immunoglobulin
IQR: interquartile range
NNT: number needed to treat
SNSD: serious neurological sequelae or death
