# Supplementary figures and images for: Prenatal Treatment for Serious Neurological Sequelae of Congenital Toxoplasmosis: An Observational Prospective Cohort Study
Source: PLoS Med. 2010 Oct 12;7(10):e1000351. doi: 10.1371/journal.pmed.1000351 (PMC2953528; doi:10.1371/journal.pmed.1000351)

Figure S1

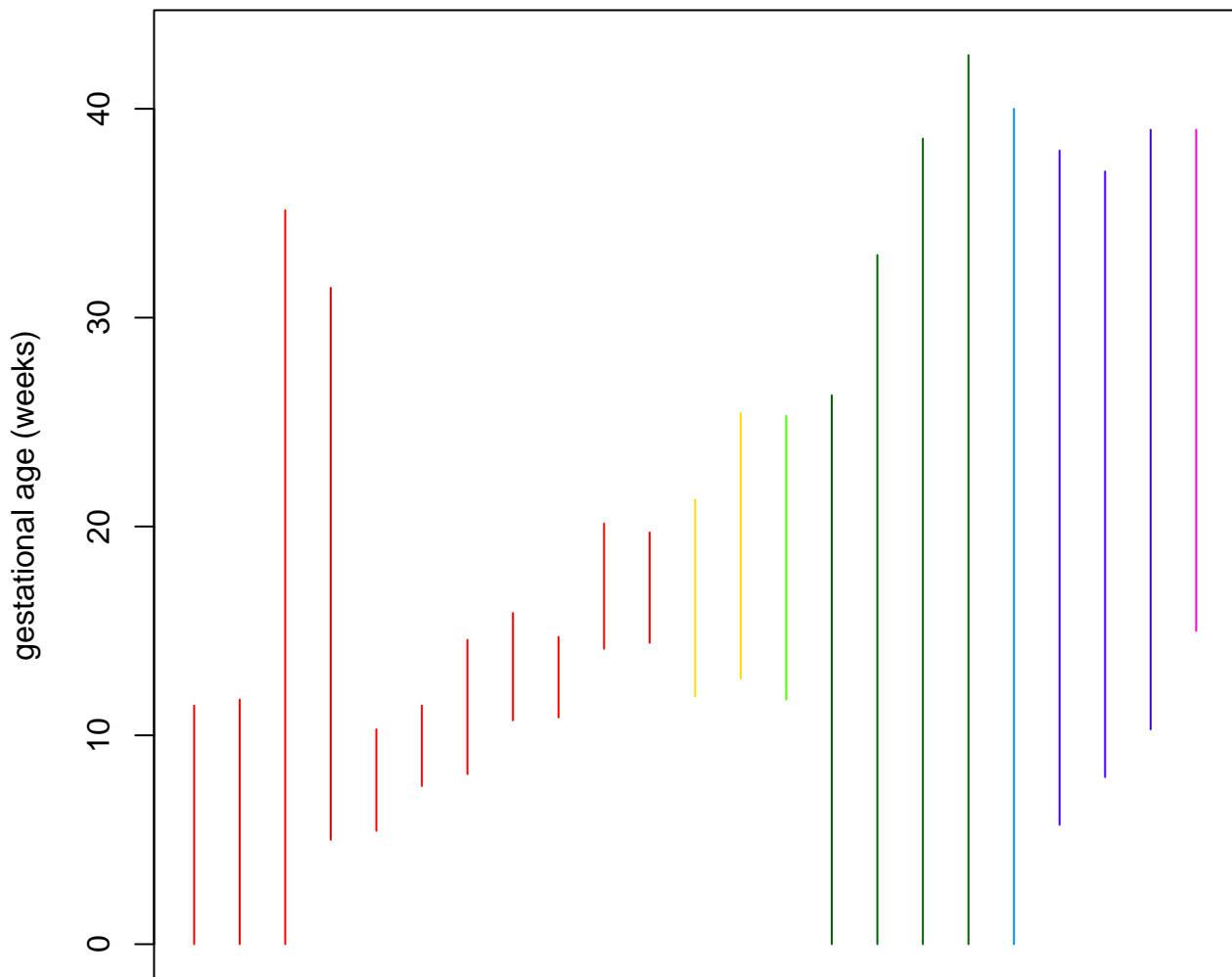

Supplement: Figure S1 — Interval for gestational age at seroconversion for pregnancies affected by SNSD. The vertical lines show the GASC interval for each pregnancy affected by SNSD (n = 23). Colours denote country of birth: red, France; yellow, Italy; light green, Austria; dark green, Poland; light blue, Denmark 1997–2000; purple, Denmark 1992–1996; pink, Sweden. (0.01 MB PDF) [file pmed.1000351.s001.pdf]

Figure S2

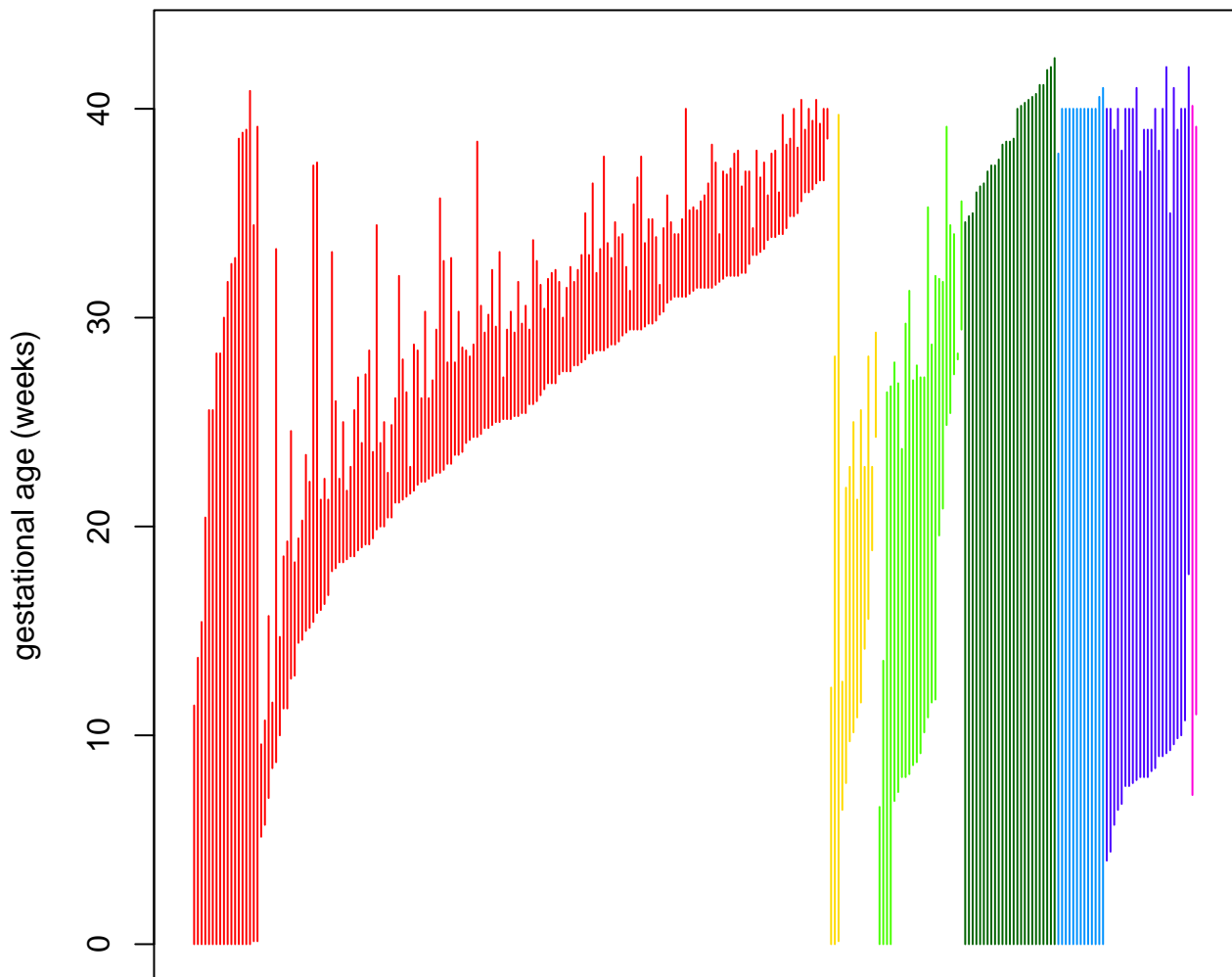

Supplement: Figure S2 — Interval for gestational age at seroconversion for unaffected pregnancies. The vertical lines show the GASC interval for each unaffected pregnancy (n = 270). Colour codes as for Figure S1. (0.01 MB PDF) [file pmed.1000351.s002.pdf]
